# A Retrospective Analysis on the Influence of Gender in the Presentation and Outcomes of Surgical Thromboembolectomy for Treatment of Acute Lower Limb Ischemia

**DOI:** 10.3390/jcm14041122

**Published:** 2025-02-10

**Authors:** Antonio Casagrande, Giulia Moretti, Beatrice Grando, Cristiano Calvagna, Giovanni Badalamenti, Filippo Griselli, Antonino Loggiacco, Sandro Lepidi, Mario D’Oria

**Affiliations:** 1 Division of Vascular and Endovascular Surgery, Cardio-Thoracic-Vascular Department, Integrated University Healthcare Giuliano-Isontina, University Hospital of Cattinara, 34149 Trieste, Italy; 2 Division of Vascular and Endovascular Surgery, Department of Clinical Surgical and Health Sciences, University of Trieste, 34149 Trieste, Italy

**Keywords:** acute limb ischemia, sex, surgical thromboembolectomy, Fogarty catheter, lower limb

## Abstract

**Background/Objectives:** We aim to quantify the effect of sex upon the presentation of acute lower limb ischemia (ALI) and the outcomes after surgical thromboembolectomy with a Fogarty catheter. **Methods**: This was a monocentric retrospective observational study of ALI treated by a Fogarty catheter. Demographics, comorbidities, and clinical characteristics were analyzed. The logistic regressions were used to estimate mortality and secondary outcomes. **Results:** Over 8 years, 193 patients (79 males and 114 females) underwent Fogarty catheter thromboembolectomy to treat acute lower limb ischemia. Females were older (74.5 for females vs. 82.5 for males) and more affected by congestive heart failure (27% vs. 8%; *p* = 0.001) and atrial fibrillation (68% vs. 37%; *p* = <0.001) than the male counterparts. Regarding etiology (*p* < 0.001), a cardiac embolism (males: 35%; females: 67%) and aortic thrombosis (males: 1%; females: 8%) were more associated with the female gender in the development of acute lower limb ischemia, while vascular bypass/endograft failure (males: 20%; females: 7%) and iatrogenic complications (males: 13%; females: 1%) were more associated with the male gender. After 30 days from surgery, 19% of men and 32% of women had died. Males had higher rates of loss of vascular patency (males: 25%; females: 9%; *p* = 0.002) and vascular reintervention (males: 20%; females: 8%; *p* = 0.012). After 90 days from surgery, 23% of men and 41% of women had died. If women had higher mortality (males: 23%; females: 41%; *p* = 0.008), men had higher rates of loss of vascular patency (males: 27%; females: 12%; *p* = 0.011) and vascular reintervention (males: 23%; females: 9%; *p* = 0.007). **Conclusions:** Older females with atrial fibrillation and/or chronic heart failure are at high risk for ALI. Regarding the thromboembolectomy with a Fogarty catheter, male sex appears to be a risk factor (OR: 2.2, CI: 1.08–4.56) for loss of primary patency, major amputation, and new vascular surgery. A further prospective analysis is warranted to understand the impact of sex in the presentation of acute lower limb ischemia (ALI) and the outcome after surgical thromboembolectomy.

## 1. Introduction

Acute lower limb ischemia (ALLI) is defined as a sudden decrease in perfusion to the lower limb that threatens its viability, thereby requiring urgent evaluation and treatment [1,2]. This condition represents a medical emergency as it carries risks of amputation and death in up to 15–20% of cases within thirty days of revascularization [3].

From a clinical standpoint, the disease is characterized by Pratt’s 5 Ps: pallor, pain, pulselessness, paresthesia, paralysis, and poikilothermia [4]. However, clinical presentation often does not include all these signs and symptoms, and a high index of suspicion is needed. In fact, the 2020 European Society for Vascular Surgery clinical practice guidelines on ALLI state that the actual incidence of the pathology cannot be clearly defined because the variety of clinical presentations makes case selection for epidemiological studies complicated [5].

Nowadays, there are several treatment approaches available for revascularization, including both open surgery techniques (such as surgical embolectomy and vascular bypass) and endovascular procedures (such as catheter-directed thrombolysis and percutaneous thromboaspiration). Among these, surgical embolectomy is the most commonly used open surgical technique and has long been the gold standard [6].

Recently, in the field of vascular surgery, several studies have identified gender-related differences in the presentation of various pathologies and in the effectiveness of different types of treatments. For example, men over 65 years of age have shown a prevalence of an abdominal aortic aneurysm approximately three times higher than women of the same age [7,8], while fatal ruptures are three times more frequent in females [9]. Similarly, other studies have shown worse outcomes after repair of abdominal aortic aneurysms in females as compared with females. However, as of the time of writing this paper, the literature still lacks robust scientific studies focused on investigating sex-related differences in the presentation and outcomes of ALLI. Therefore, the objective of this study was to verify, in the context of surgical embolectomy for the treatment of ALLI, the potential prognostic impact of gender-related features on the clinical features and outcomes of ALLI. Finding different outcomes following surgical intervention for ALLI in one of the two sexes could prompt a reassessment of therapeutic strategies and the personalization of surgical treatment.

## 2. Methods

### 2.1. Study Design

A single-center retrospective observational study was conducted, reviewing the clinical data for consecutive patients with ALLI who underwent Fogarty catheter thromboembolectomy (with or without adjuvant open or endovascular procedures) between 1 January 2016 and 31 December 2023. The study was approved by the ethical committee of the treating institution and the need for informed consent was waived considering the retrospective nature with minimal risk to study subjects. Exclusion criteria for the study included patients who developed ALLI with a previous diagnosis of chronic limb-threatening ischemia (*n* = 32), patients with ALLI treated endovascularly with percutaneous aspiration (*n* = 15), patients with ALLI from blunt or penetrating non-iatrogenic trauma (*n* = 8), patients with ALLI from a thrombosed popliteal aneurysm or acute aortic dissection (*n* = 15), or inability to access the patient’s medical records for retrieval of comprehensive documentation on primary outcomes (*n* = 0).

The study cohort was stratified in two groups for all analyses, based on sex (i.e., males vs. females). Technical success was defined as the effective restoration of perfusion in the femoro-popliteal segment with at least one patent tibial artery and decrease in Rutherford class ≥ 1 as compared with preoperative status. The primary outcome of the study was binary all-cause mortality. As for secondary outcomes, we evaluated a binary composite of loss of patency, new vascular intervention, or major limb amputation (MLA) as well as time-to-event long-term survival. Where possible, postoperative outcomes were evaluated at 30 days and 90 days. Long-term survival was first evaluated in the full cohort (i.e., including early deaths), as well as in the restricted cohort of patients who were alive for at least thirty days following surgical intervention (to provide a sensitivity analysis that could account for the expected high rate of early postoperative mortality). The survival analysis was censored as of 31 December 2024.

### 2.2. Statistical Analysis

For each continuous variable, the mean and standard deviation were calculated for the two groups after checking for normal distribution. Categorical or binary variables were reported as absolute counts and percentages. Subsequently, a two-tailed *t*-test or chi-square test was used to compute the *p*-value (Fisher’s exact test was applied if the count of positive or negative cases in a group for a given variable was less than five). The time-to-event analysis was conducted according to the Kaplan–Meier method, with a log-rank test for estimation between groups. In all cases, the significance level α was set at 0.05.

Binary logistic regression models were created to evaluate associations between the outcomes of interest at 90 days with independent variables. The variables for the model were selected based on a univariate analysis. The results obtained were expressed as odds ratios (ORs) with their 95% confidence intervals (CIs). For long-term survival estimates, the Cox Proportional Hazard regression was used to assess for independent associations between the outcomes of interest and independent variables. The results obtained were expressed as hazard ratios (HRs) with their 95% confidence intervals (CIs). The statistical analysis was performed using the open-source R software (version 4.3.3. with R Commander package).

## 3. Results

### 3.1. Study Sample

**Figure 1:**
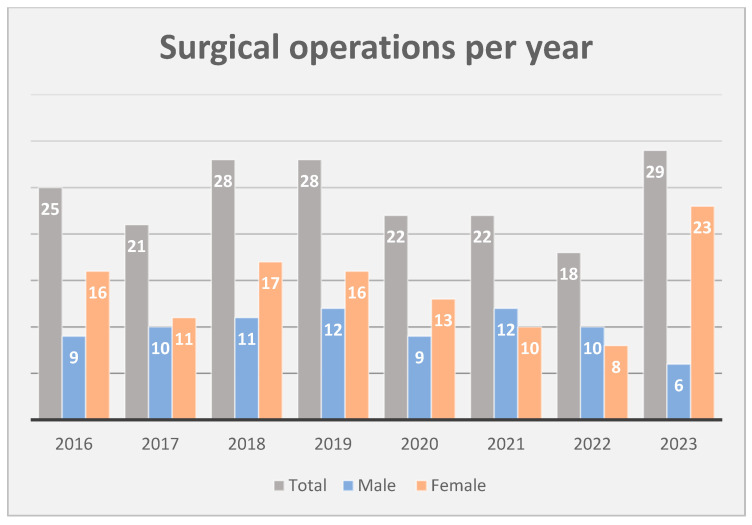
Trend over time depicting overall number of acute limb ischemia cases, with proportion of males and females.

A total of 193 consecutive patients (79 males and 114 females) who underwent Fogarty catheter thromboembolectomy to treat ALLI during the study period were identified and included in the final analysis. Specifically, the procedures were distributed over the 8 years of study as depicted in Figure 1.

### 3.2. Medical History

Regarding the medical history (Table 1), the average age of patients at the time of the procedure was 74.5 years (σ = 13) for males and 82.5 years (σ = 10) for females (*p* < 0.001). Among all the variables considered, those with the highest prevalence in the patient sample were hypertension (71% in males vs. 78% in females; *p* = 0.397), dyslipidemia (52% in males vs. 43% in females; *p* = 0.163), atrial fibrillation/flutter (37% in males vs. 68% in females; *p* < 0.001), diabetes (32% in males vs. 25% in females; *p* = 0.29), and congestive heart failure (8% in males vs. 26% in females; *p* = 0.001). Males also had significantly lower CHA2DS2-VASc scores as compared with females (mean: 2 vs. 4 points, *p* < 0.001).

### 3.3. PreOperative Details

Regarding the preoperative details (Table 2), the most frequent etiology was a cardiac embolism, identified as the cause of ALLI in 35% of males and 67% of females (*p* < 0.001). Conversely, failure of a prior graft/endograft or iatrogenic complications as the underlying etiology of ALLI were more frequent in males (20% and 13%, respectively) as compared with females (7.02% and 0.88%, respectively).

Most commonly, patients presented for medical attention with a Rutherford grade of IIa (40% of males and 35% of females) or IIb (39% of males and 39% of females), without statistically significant differences between groups (*p* = 0.809). Computed tomography angiography (53% of males and 41% of females), followed by duplex ultrasound (32% of males and 46% of females), was the most commonly used imaging modality, without statistically significant differences between groups (*p* = 0.068).

### 3.4. Surgical Procedure

Regarding the surgical procedure (Table 3), only thromboembolectomy with a Fogarty catheter was required in most cases: indeed, in a few cases, another type of intervention was performed, either open (males: 13%; females: 10%; *p* = 0.711) or endovascular (males: 27%; females: 24%; *p* = 0.522). Nevertheless, in 66% of males and 62% of females, the procedure was able to restore patency to at least two different tibial vessels in almost two thirds of cases (males: 66% vs. females: 6%; *p* = 0.693). Surgery lasted, on average, 123 min (σ = 83) for males and 105 min (σ = 71) for females (*p* = 0.125) and was clinically effective in 85% of males and 83% of females (*p* = 0.730).

### 3.5. Outcomes at 30 Days

Regarding the primary outcome at 30 days after the procedure (Table 4A), 19% of men and 32% of women who underwent surgical thromboembolectomy were deceased (*p* = 0.041). However, regarding the secondary outcomes, there were more instances of loss of primary patency (males: 25%; females: 9%; *p* = 0.002), major amputation (males: 16%; females: 9%; *p* = 0.005), and vascular reintervention (males: 20%; females: 8%; *p* = 0.012) in males as compared with females. No statistically significant differences were found in the average length of stay in the intensive care unit as well as in the total mean duration of hospitalization. 

### 3.6. Outcomes at 90 Days

Regarding the primary outcome at 90 days after the procedure (Table 4B), 23% of men and 41% of women had died (*p* = 0.008). However, regarding the secondary outcomes, the male gender still had higher rates of loss of primary patency (males: 27%; females: 12%; *p* = 0.011), major amputation (males: 18%; females: 10%; *p* = 0.15), and vascular intervention (males: 23%; females: 9%; *p* = 0.007).

The logistic regression model for the primary outcome (i.e., 90-day mortality) showed a statistically significant association between age and the probability of death at 90 days (OR: 1.05; 95% CI: 1.01–1.1; *p* = 0.02), while no associations were found with gender (Table 5A). Additionally, having at least two patent leg vessels after surgery was shown to be negatively associated with early death (OR: 0.26; 95% CI: 0.17–0.58; *p* = 0.001).

Looking at the secondary outcome (i.e., occurrence within 90 days of at least one out of loss of patency, major amputation, and new vascular intervention), the logistic regression model showed a statistically significant association between female gender and the risk of the secondary outcomes (OR: 2.21; 95% CI: 1.08–4.56; *p* = 0.038). Again, having at least two patent leg vessels after surgery was shown to be negatively associated with the secondary outcome (OR: 0.18; 95% CI: 0.05–0.56; *p* = 0.001).

### 3.7. Long-Term Survival

**Figure 2:**
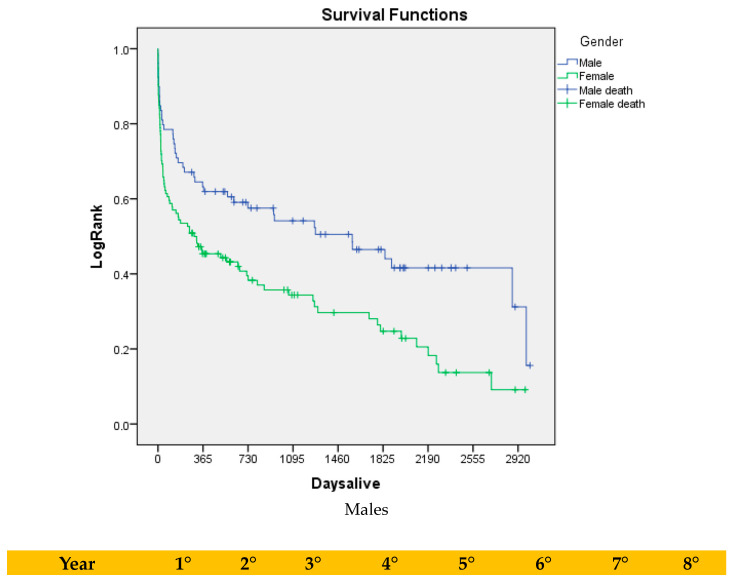

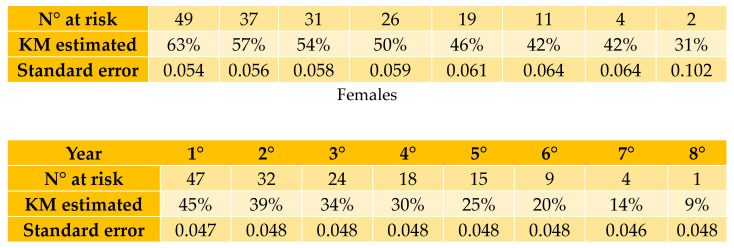
Kaplan–Meier analysis of long-term survival in full cohort (i.e., including 30-day mortality events) stratified in two groups based on gender.

**Figure 3:**
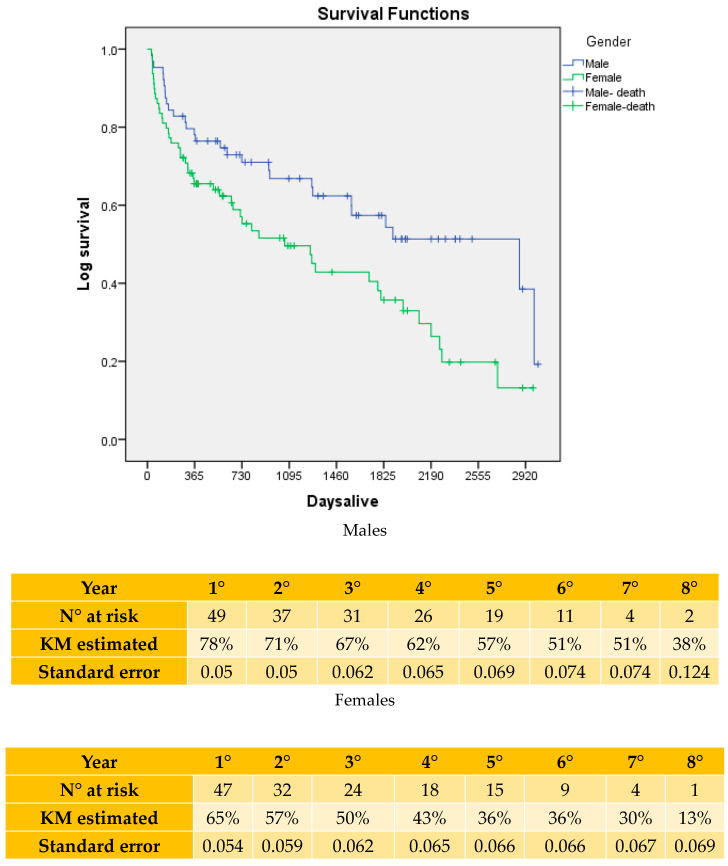
Kaplan–Meier analysis of long-term survival in restricted cohort (i.e., after excluding 30-day mortality events) in the study population stratified in two groups based on gender.

At five years, the estimated survival in the full cohort was 46% for males as compared with 25% for females (*p* = 0.002; Figure 2). In the sensitivity analysis, after accounting for early (i.e., within 30 days of index operation) deaths, the long-term survival rates were still significantly higher in males as compared with females (57% vs. 36%; *p* = 0.01; Figure 3). However, Cox Proportional Hazards did not identify female gender to be associated with increased risk for long-term death (HR: 1.156; 95%CI: 0.833–1.872; *p* = 0.28). In contrast, increasing age was indeed associated with higher hazards for long-term mortality (HR: 1.148; 95%CI: 1.028–1.368; *p* = −001).

## 4. Discussion

### 4.1. Summary of Findings and Comparison with Previous Research

In this contemporary single-center investigation, we have shown that ALLI remains burdened with significant life-threatening and limb-threatening events, especially early after surgery. In fact, in our cohort, mortality and amputation rates at 90 days were between 20% and 40% (for males and females, respectively) and between 17% and 10% (for males and females, respectively). However, our findings also indicate some notable differences in baseline presentation between genders that may drive outcomes and offer options for personalized care. For instance, the average age of male patients in the study was significantly lower than for the female ones, a finding which has several diagnostic and prognostic implications. Indeed, we believe that this could be linked, for instance, to the different prevalent etiology of ALLI in males as compared with females (with the latter being more often affected by a cardiac embolism), as well as to the lower five-year survival in women as compared with men even after performing an ad hoc sensitivity analysis excluding perioperative death events.

Chihade et al., in a multicenter study from 2023 comparing outcomes between the two sexes after revascularization interventions, reported an average age of 65.8 ± 14.8 years for males and 64 ± 13.3 years for females (*p* < 0.001) [10]. Unfortunately, we could not explain why our study includes patients who are, on average, ten years older than those in Chihade’s study. However, both studies clearly indicate that women may tend to develop acute ischemia later than men. While the literature on chronic ischemic disease often attributes this delay to the protective role of estrogen [11] and/or a less evident symptomatology, the data collected in this study on acute pathology suggest that this delay is related to the different etiologies prevalent in the two sexes.

Cardiac pathology, whether atrial fibrillation/flutter or congestive heart failure, is certainly the most common element in the medical history of the patients in this study, with a higher and statistically significant incidence in females. Considering that a cardiac embolism was identified as the etiological cause in almost two thirds of females, this aligns with several studies in the literature that have identified the female gender as an independent risk factor for the development of systemic thromboembolic events and stroke related to atrial fibrillation [12,13,14]. Unlike the female cohort, the male patients in our study did not show such a predominant etiology, with cardiac embolisms in only about one third of cases. Most importantly, females were most likely to be in higher CHA2DS2-VASc risk categories than males (a finding that was consistent with their higher average age), with only 2% of this group of patients having a score < 2, which is usually regarded as the threshold for initiating oral anticoagulation. While we were unable to assess the precise causes as to why many females were not on chronic anticoagulation, it is likely that their physicians or cardiologists might have withdrawn treatment because of high estimated bleeding risk. However, our study highlights the need for careful balance of the risk–benefit of chronic anticoagulation given the not-benign nature of ALLI.

Regarding the outcomes of surgical thromboembolectomy at 30 days after the surgery, the mortality rate in the female population (almost 32%) was higher than in the male counterparts (less than 20%). On the other hand, males showed a greater tendency to lose the vascular patency achieved after the surgery and to require a new vascular intervention compared to females. Indeed, the higher incidence of failure of previous vascular interventions as the underlying etiology of ALLI in males may explain why they were subjected to higher rates of loss of primary patency. These results are in line with those obtained by Chihade et al. [10]. However, the literature still needs to explain whether the increased mortality in the female cohort is solely due to gender, the older age of women in the sample, or a combination of both [15]. Unlike the literature, in this study, female sex was not identified as an independent risk factor for 30-day mortality. Indeed, the results of the logistic regression model highlight that only age constitutes an independent risk factor for 30-day mortality. Therefore, it can be stated that the increased mortality in the female population is likely due to the higher average age of women rather than to gender itself. The older age may also explain why females exhibited decreased life expectancy in the long run, with gender not being independently associated with long-term mortality in the Cox models. Therefore, these findings seem to highlight the role of gender-related explanatory variables rather than gender itself as key factors that drive clinical outcomes.

### 4.2. Implications of the Results and Directions for Future Research

ALLI represents a medical and surgical emergency due to its high risk of limb loss and death. Unfortunately, evaluating the outcomes of this condition remains difficult and complex due to differences in patient characteristics, symptomatic presentation, and therapeutic approaches. Nonetheless, this study has revealed significant trends, which are also confirmed by previous research, highlighting a higher incidence of secondary limb-related adverse events in males following surgical thromboembolectomy. Conversely, the difference in short-term and long-term mortality (against women) appears to be attributed to the age disparity between the two groups. Moreover, the significant prevalence of a cardiac embolism as the primary etiological cause in females may suggest the need for more frequent cardiovascular monitoring or better anticoagulation management for these elderly patients. However, to fully understand the impact of gender on the presentation of ALLI and the outcomes following thromboembolectomy with a Fogarty catheter, it is essential to conduct targeted and in-depth future research. Therefore, future studies should focus on the detailed analysis of a broader range of variables in a much larger patient sample than the one considered in this study, providing a more comprehensive and detailed view on how gender influences this critical vascular condition.

### 4.3. Strengths and Limitations of the Study

The strengths of this research are the homogeneous sample of patients, all of whom underwent Fogarty catheter thromboembolectomy (with possible adjunctive endovascular interventions) for ALLI at a single center by an experienced group of vascular/endovascular surgeons, the wide range of baseline characteristics considered for each patient with a minimal amount of missing data (<2% for all considered variables), and a consistent long-term follow-up to monitor the occurrence of relevant outcomes. In this regard, this scientific research represents an original and innovative contribution to the field of vascular surgery.

The limitations of this study are mainly the limited sample size and the retrospective design, which may influence the presence of unmeasured confounders, although we attempted to correct for known ones using robust regression analytical techniques. Furthermore, laboratory values were not available for retrospective review as well as some details that could have been used for estimating risk of bleeding by the calculation of previously published scores.

## 5. Conclusions

In this contemporary single-center investigation, ALLI remains burdened with high rates of postoperative mortality and limb-related morbidity. Notably, there appears to be significant differences in the main etiologies of ALLI between males and females, which may be important for targeting gender-specific preventive approaches to mitigate ALLI risk. Furthermore, such differences may explain the different rates of limb-related morbidity, which appeared as higher in males but not independently related to gender. In contrast, females appeared to be older at the time of index presentation, which may be the reason behind the observed higher rates of mortality in the short term as well as in the long term. Future research is needed to fill the knowledge gaps regarding gender-specific differences in causes and treatment of ALLI, conducting comprehensive studies that will explore these disparities to develop targeted prevention algorithms and management strategies. By addressing these gaps, healthcare providers can improve clinical outcomes and ensure that both men and women receive optimal care tailored to their specific characteristics and needs.

## Figures and Tables

**Table 1:** Baseline demographics and medical comorbidities of the study population stratified in two groups based on gender. Bold marks *p* values that are statistically significant.

	Males (*n* = 79)	Females (*n* = 114)	*p*-Value
**Age in years (Mean + SD)**	74 (13)	82 (10)	**<0.001**
**Age groups (%)**			
≥80 years	33 (42)	78 (68)	**<0.001**
70–80 years	22 (28)	23 (20)	0.215
≤70 years	24 (30)	13 (11)	**<0.001**
**Diabetes (%)**	25 (32)	29 (25)	0.290
**Hypertension (%)**	56 (71)	89 (78)	0.397
**Coronary artery disease (%)**	17 (22)	17 (15)	0.204
**Congestive heart failure (%)**	6 (8)	30 (26)	**0.001**
**Active smoker (%)**	18 (23)	16 (14)	0.098
**Severe chronic kidney disease with eGFR < 30 (%)**	13 (16)	29 (25)	0.161
**Previous cerebrovascular accident (%)**	5 (6)	12 (10)	0.255
**Hyperlipidemia (%)**	41 (52)	49 (43)	0.163
**Active cancer (%)**	13 (16)	10 (9)	0.091
**Chronic obstructive pulmonary disease (%)**	10 (13)	14 (12)	0.885
**Obesity (%)**	10 (13)	20 (18)	0.396
**Atrial fibrillation/flutter (%)**	29 (37)	78 (68)	**<0.001**
**CHA2DS2-VASc (Mean + SD)**	2.76 (1.37)	4.43 (1.30)	**<0.001**
**CHA2DS2-VASc groups (%)**			**<0.001**
**0–1**	16 (21)	3 (2)	
**2–5**	62 (78)	92 (81)	
**6–9**	1 (1)	19 (17)	
**COVID-19 positivity within 30 days of index procedure (%)**	8 (10)	11 (10)	0.913

**Table 2:** Etiology and severity of acute limb ischemia, preoperative antithrombotic therapy, and type of preoperative imaging. Bold marks *p* values that are statistically significant.

	Males (*n* = 79)	Females (*n* = 114)	*p*-Value
**Etiology:**			**<0.001**
Cardiac embolism (%)	47 (59)	91 (80)	
Non-cardiac embolism (%)	4 (5)	5 (4)	
Failure of prior graft or endograft (%)	16 (20)	8 (7)	
Aortic thrombosis (%)	2 (3)	9 (8)	
Iatrogenic complication (%)	10 (13)	1 (1)	
**Preoperative therapy:**			0.115
None (%)	32 (41)	34 (30)	
Single antiplatelet therapy (%)	23 (29)	45 (39)	
Dual antiplatelet therapy (%)	7 (9)	2 (2)	
Vitamin K antagonists (%)	6 (8)	11 (10)	
Direct oral anticoagulants (%)	8 (10)	19 (17)	
Antiplatelet + anticoagulant (%)	3 (4)	3 (3)	
**Rutherford score:**			0.809
1 (%)	6 (8)	12 (11)	
2a (%)	32 (41)	40 (35)	
2b (%)	31 (39)	45 (39)	
3 (%)	10 (13)	17 (15)	
**Preoperative imaging:**			0.068
Nothing (%)	8 (10)	14 (12)	
Duplex ultrasound (%)	25 (32)	52 (46)	
Computed tomography angiography (%)	42 (53)	47 (41)	
Catheter-based angiography (%)	4 (5)	1 (1)	
**Ischemia time > 6 h (%)**	52 (66)	77 (68)	0.803

**Table 3:** Intraoperative details and early postoperative outcomes of surgical procedures for treatment of acute limb ischemia in the study population.

	Males (*n* = 79)	Females (*n* = 114)	*p*-Value
**Additional open procedure:**			0.711
None (%)	69 (87)	102 (89)	
Patch/endarterectomy (%)	10 (13)	12 (11)	
**Additional endovascular procedure:**			0.522
None (%)	59 (75)	87 (76)	
Proximal PTA/stenting (%)	5 (6)	9 (8)	
Distal PTA/stenting (%)	12 (15)	17 (15)	
Both proximal and distal PTA/stenting (%)	3 (4)	1 (1)	
**Outflow vessels at end of procedure ≥ 2 (%)**	52 (66)	71 (62)	0.693
**Final control at end of procedure:**			0.327
Clinical (%)	4 (5)	7 (6)	
DUS (%)	45 (60)	72 (63)	
Angiography (%)	30 (38)	35 (31)	
**Intraoperative fasciotomy (%)**	4 (5)	2 (2)	0.229
**Procedure time in minutes (Mean + SD)**	123 (83)	106 (71)	0.125
**Technical success (%)**	70 (89)	102 (89)	0.631
**Postoperative fasciotomy (%)**	1 (1)	2 (2)	1
**Postoperative antithrombotic therapy:**			0.730
Low-Molecular-Weight Heparin (%)	21 (27)	41 (36)	
Single antiplatelet therapy (%)	26 (33)	25 (22)	
Dual antiplatelet therapy (%)	4 (5)	5 (4)	
Vitamin K antagonists (%)	7 (9)	12 (11)	
Direct oral anticoagulants (%)	8 (10)	11 (10)	
Antiplatelet + anticoagulant (%)	11 (13)	14 (12)	

**Table 4:** Clinical outcomes after 30 days (A) and after 90 days (B) following surgical treatment for acute limb ischemia in the study population. Bold marks *p* values that are statistically significant.

A. At 30 days			
	Males (*n* = 79)	Females (*n* = 114)	*p*-Value
**Death (%)**	15 (19)	36 (32)	**0.041**
**Loss of primary patency (%)**	20 (25)	10 (9)	**0.002**
**Major amputation (%)**	13 (16)	10 (9)	**0.005**
**Vascular reintervention (%)**	16 (20)	9 (8)	**0.012**
**Blood product transfusion (%)**	20 (25)	39 (34)	0.187
**Wound infections:**			0.348
Superficial (%)	3 (4)	9 (8)	
Profound (%)	5 (6)	4 (4)	
**Days in intensive care unit (Mean + SD)**	1 (5)	2 (7)	0.664
**≥5 days in ICU (%)**	6 (8)	9 (8)	0.939
**Days in hospital (Mean + SD)**	17 (23)	13 (18)	0.202
**≥10 days in hospital (%)**	34 (43)	44 (39)	0.536
**B. At 90 days**			
	**Males** **(*n* = 79)**	**Females** **(*n* = 114)**	***p*-Value**
**Death (%)**	18 (23)	47 (41)	**0.008**
**Loss of primary patency (%)**	21 (27)	14 (12)	**0.011**
**Major amputation (%)**	14 (18)	12 (11)	0.15
**Vascular reintervention (%)**	18 (23)	10 (9)	**0.007**

**Table 5:** Logistic regression analysis for independent predictors of primary outcome (mortality at 90 days after index procedure) and secondary outcome (composite of loss of primary patency, major amputation, or vascular reintervention at 90 days after index procedure).

A. Primary Outcome			
Variable	Odds Ratio	Lower 95% CI	Higher 95% CI
**Gender (ref—male)**	1.288	0.449	3.698
**Age**	1.051	1.001	1.112
**AF/flutter**	2.058	0.742	6.126
**Chronic heart failure**	2.063	0.689	6.221
**Rutherford score ≥ 2b**	0.830	0.328	2.021
**Ischemia time > 6 h**	1.224	0.489	3.211
**Outflow vessels (ref ≥ 2 vessels)**	0.225	0.081	0.593
**B. Secondary Outcome**			
**Variable**	**Odds Ratio**	**Lower 95% CI**	**Higher 95% CI**
**Gender (ref—male)**	2.207	1.086	4.559
**Age**	0.990	0.951	1.030
**AF/flutter**	0.247	0.062	0.855
**Chronic heart failure**	0.574	0.0286	3.935
**Rutherford score ≥ 2b**	0.521	0.163	1.528
**Ischemia time > 6 h**	1.549	0.522	5.027
**Outflow vessels (ref ≥ 2 vessels)**	0.183	0.054	0.568

## Data Availability

Data can be made available by motivated request to the corresponding author and are stored in a secure database.
